# Inequalities in out-of-pocket health expenditure among women of reproductive age: after-effects of national health insurance scheme initiation in Ghana

**DOI:** 10.1186/s42506-020-00064-9

**Published:** 2021-03-11

**Authors:** Michael Ekholuenetale, Amadou Barrow

**Affiliations:** 1 Department of Epidemiology and Medical Statistics, Faculty of Public Health, College of Medicine, University of Ibadan, Ibadan, Nigeria; 2 Department of Public & Environmental Health, School of Medicine & Allied Health Sciences, University of The Gambia, Kanifing, Banjul, The Gambia

**Keywords:** SES, Maternal health, Lorenz curves, Concentration Index, Women

## Abstract

**Background:**

Improvement in maternal healthcare is a public health priority. Unfortunately, in spite of the efforts made over time regarding universal coverage, there remain issues with accessibility and use of healthcare services up to now. In this study, we examined inequalities in out-of-pocket health expenditure among women of reproductive age in Ghana. We analyzed secondary data collected in Ghana Demographic and Health Survey (GDHS) - 2014. A total of 9,002 women of reproductive age were included in this study. Lorenz curves and the concentration index were used to examine neighborhood socioeconomic disadvantage inequalities in out-of-pocket expenditure for maternal healthcare utilization

**Results:**

About two thirds (66.0%) of women of reproductive age in Ghana were covered by health insurance. In sum, women of high neighborhood socioeconomic disadvantage status had the least out-of-pocket expenditure for total healthcare utilization, laboratory investigations, antenatal care visits, post-natal care visits, care for new born for up to 3 months, and other healthcare services. The converse was however true for family planning service utilization. Using Concentration Index, we quantified the degree of neighborhood socioeconomic disadvantage inequalities in healthcare service utilizations.

**Conclusion:**

This study showed a gap in health insurance coverage among women of reproductive age. There were also inequalities in out-of-pocket expenditure for healthcare services utilization. It is expedient for stakeholders in the healthcare system to make policies targeted at bridging the neighborhood socioeconomic differences in maternal healthcare use and develop programs to improve women’s financial protection. Moreover, enlightenment on health insurance availability and coverage should focus on women at risk of out-of-pocket expenditure.

## Background

The United Nations Sustainable Development Goals (SDGs) have prioritized protection against financial risk for healthcare costs through reinforcement of national policies for both developed and developing countries. In addition to geo-spatial access, financial accessibility to vital healthcare has become a significant condition to achieve the universal health coverage (UHC) [1, 2]. It was reported that more than 150 million people experience financial catastrophe, while approximately 100 million are sent into poverty annually due to practical out-of-pocket healthcare expenditure worldwide [3]. Out-of-pocket health expenditure has adverse effects on family income allocation to essential needs, including food, education, shelter, utilities, and so forth [4, 5]. Consequently, there is a need for healthcare systems to establish financial protection for the populace against the burden of health problems. It is against this backdrop that the World Health Assembly resolution 58.33 requested member states to target achievement of the UHC [6], through equality in access to healthcare services utilizations especially when required with no financial barriers. This involves multi-facets strategies including the proportion of health costs covered, the range of services covered, and the proportion of people covered [2].

In Ghana, the National Health Insurance Scheme (NHIS) was initiated as a major tool to eliminate barriers in accessing healthcare services. The National Health Insurance Act (Act 650) scheme was finalized in 2003 with the aim of enhancing financial access, particularly among the key population for quality healthcare utilization [7]. NHIS is a scheme renewable every year and based on contributions from clients, while cardholders will access the health facilities certified by the National Health Insurance Authority [8]. The structure of the contribution is such that clients pay based on their income, while individuals receive in accordance with their needs. By doing so, health insurance subsidizes the health cost for clients and the well-off pays for members of his/her household, including the aged and needy [9].

Healthcare financing has gone through several turns and twists in Ghana. Before the country’s independence in 1957, health financing was prominently through out-of-pocket expenditure. The effect of out-of-pocket health expenditure made Ghana a foremost country in sub-Sahara Africa to adopt the NHIS to reduce the plight of her citizens [4, 7]. Notwithstanding, an individual can be registered for health insurance and yet not covered; by implication, one can have a valid insurance card and still unable to access all available services from health insurance scheme [10]. For example, if an individual is yet to pay his premium in full, or if he is in the waiting period between the completion of registration paperwork, then s/he may be registered and be partially covered or not covered at all. Though more than 70% of women have registered with health insurance schemes in the country, nonetheless, less than half are eventually covered by an insurance scheme [11], whereas approximately 20% of them are not registered nor covered under any scheme. In Ghana, NHIS remains the prominent type of health insurance scheme for women’s healthcare [11].

Generally, providing the populace with universal access to healthcare services is a key strategic intervention for promoting socioeconomic advancement [1, 12]. Until now, developing and implementing effective healthcare-financing approach instigates debates among policy- and decision-makers and public health professionals, specifically in resource-constrained settings [1, 2, 13], where healthcare systems are commonly under-funded [14]. In line with Structural Adjustment Programs in the 1980s, several poor-resource settings added user fees at the point of healthcare service delivery for fund raising and resource mobilization for their health systems [15–17].

While such user fees have been a vital channel of fund raising for healthcare providers and governments, they have also been the root cause for inaccessibility to healthcare services, especially among the disadvantaged [1, 14, 18]. Considering the problems linked with requesting user fees at the point of health service delivery and how it has deprived people from accessing healthcare, in recent times, the international community supports Social Health Insurance (SHI) as a tool for eliminating financial barriers to healthcare utilization, especially in resource-constrained settings [6]. Besides improving the accessibility and utilization of health facilities by the populace, health insurance scheme is a method of fund raising and resource mobilization for health service providers, which makes the entire system sustainable [1, 6]. Considering the above, we examined inequalities in out-of-pocket health expenditures among women of reproductive age in Ghana. The findings will add to the literature on health financing, as there is a paucity of data that examined NHIS utilization across socioeconomic disadvantaged groups.

## Methods

### Data source

In this study, secondary data analysis was conducted using the 2014 GDHS. A total of 9002 women of reproductive age were included in this study. DHSs are designed to collect data on family planning, child health, HIV/AIDS, and sexual and reproductive health. Based on the topic of the survey, usually women of reproductive age are the target of the survey. Women who meet the eligibility criteria for the individual woman interview are identified from the selection of households. Hence, all DHS use at least two questionnaires, for example, Women’s Questionnaire and a Household Questionnaire. DHS data are publicly available and can be accessed from MEASURE DHS database at http://dhsprogram.com/data/available-datasets.cfm. A multi-stage stratified cluster design is used in DHS, based on a list of enumeration areas (EAs), which are systematically selected units from localities and constitute the local government areas (LGAs). The details of DHS sampling procedure have been reported in a previous study [19].

### Outcome variable

The outcome variables were measured dichotomously (yes vs. no) as reported by the women, pay out-of-pocket for drugs and services, family planning, laboratory investigations, antenatal care, post-natal care, care for newborn up to 3 months, and other maternal healthcare services.

### Independent variables

Health insurance coverage was measured dichotomously: insured/covered vs. not insured/not covered. Household wealth quintile has been measured by DHS using principal component analysis (PCA) to assign the wealth indicator weights. This procedure assigned scores and standardized the wealth indicator variables using household assets including ownership of bicycle, car/truck, motorcycle/scooter, type of wall, floor and roof material, sanitation facilities, water sources, television, radio, electricity, cooking fuel, refrigerator, furniture, and the number of people who sleep in a room among other numerous items. The factor coefficient scores (factor loadings) and *z* scores were then calculated. For each household, the indicator values were multiplied by the loadings and summed to produce the household’s wealth index value. The standardized *z* score was then used to classify the total assigned scores to the poorest, poorer, middle, richer, and richest quintiles [20, 21]. Furthermore, neighborhood socioeconomic disadvantage was an index constructed from four variables using PCA. The variables are the proportion of women with no formal education, unemployed, rural resident, and living below the poverty level (asset index below 20% poorest quintile). A standardized score with mean 0 and standard deviation 1 was generated from this index, which we divided into quartiles. Quartile 1 represented the lowest, while quartile 4 represented the highest neighborhood socioeconomic disadvantage level [22, 23].

### Ethical consideration

In this study, we used a population-based dataset available in the public domain with no participants’ identifiers in line with ethical practice of safeguarding confidentiality. The authors sought for approval with MEASURE DHS/ICF International and permission was granted to download and use the data for the purpose of this research. Notably, DHS project obtained ethical approval from the relevant research ethics committee in Ghana, West Africa, before the survey was conducted to ensure that the protocols comply with the US Department of Health and Human Services regulations for protecting human subjects.

### Statistical analysis

We used the survey module of Stata to account for stratification, clustering, and sampling weight. The percentage and chi-square test were used for summary and bivariate analyses. Concentration Index and Lorenz curves were used to examine neighborhood socioeconomic disadvantage inequalities for out-of-pocket health expenditure. Concentration Index value is positive when the Lorenz curve is below the line of equality indicating that the burden of out-of-pocket expenses is greater among high neighborhood socioeconomic disadvantage groups. Similarly, when the concentration index value is negative, it shows that the burden of out-of-pocket is higher among low neighborhood socioeconomic disadvantage groups. The insured vs. uninsured factor was used for stratified analyses. Lorenz curves and the concentration index are suitable for the analysis of such outcome variables [24]. A statistically significant difference was set at *p* < 0.05. Data analysis was conducted using Stata Version 14 (StataCorp., College Station, TX, USA).

## Results

**Fig. 1:**
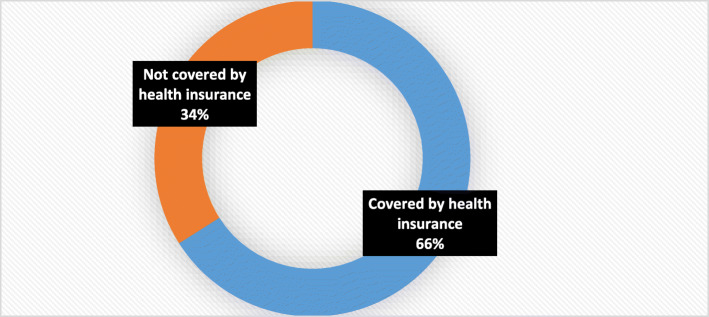
Percentage distribution of health insurance coverage of women of reproductive age, Ghana DHS, 2014

From Fig. 1, about two thirds (66.0%) of women of reproductive age in Ghana are covered by health insurance. This shows that a gap of approximately one third of women is yet to be covered by health insurance scheme (national/district health insurance).

The results from Table 1 show that approximately 32.1% of women who had out-of-pocket expenditure for drugs and services were in the lowest (quartile 1) neighborhood socioeconomic disadvantage group, whereas approximately 17.0% of women being in the highest (quartile 4) neighborhood socioeconomic disadvantage group had out-of-pocket expenditure for drugs and services. Conversely, approximately 16.9% of women who had out-of-pocket expenditure for family planning services were in the lowest (quartile 1) neighborhood socioeconomic disadvantage group; however, 32.3% of women being in the highest (quartile 4) neighborhood socioeconomic disadvantage group had out-of-pocket expenditure for family planning services. Furthermore, approximately 32.0%, 51.8%, 49.3%, 61.4%, and 30.2% of women who had out-of-pocket expenditure for laboratory investigations, antenatal care, post-natal care, care for new born up to 3 months, and other services were in the lowest (quartile 1) neighborhood socioeconomic disadvantage group, whereas 12.6%, 3.6%, 4.4%, 2.3%, and 16.1% of women being in the highest (quartile 4) neighborhood socioeconomic disadvantage group had out-of-pocket expenditure for laboratory investigations, antenatal care, post-natal care, care for new born up to 3 months, and other services, respectively.

**Table 1:** Percentage distribution of out-of-pocket expenditure on maternal healthcare by neighborhood socioeconomic disadvantage level, Ghana DHS, 2014 (*n* = 9002)

Variable	***n***	Neighborhood socioeconomic disadvantage level				***P***
		Quartile 1 (lowest)	Quartile 2	Quartile 3	Quartile 4 (highest)	
Pay out-of-pocket for drugs and services	2932	32.1	28.0	22.9	17.0	< 0.001*
Pay out-of-pocket for family planning services	260	16.9	27.7	23.1	32.3	0.002*
Pay out-of-pocket for laboratory investigations	1053	32.0	32.1	23.7	12.6	< 0.001*
Pay out-of-pocket for antenatal care	83	51.8	28.9	15.7	3.6	< 0.001*
Pay out-of-pocket for post-natal care	69	49.3	27.5	18.8	4.4	< 0.001*
Pay out-of-pocket for care for newborn up to 3 months	44	61.4	25.0	11.4	2.3	< 0.001*
Pay out-of-pocket for other services	1207	30.2	32.6	21.1	16.1	< 0.001*

*Significant at *p* < 0.05; *p* obtained using Chi-square test

In Table 2, we presented the proportion of out-of-pocket expenditure among women for overall healthcare utilization, family planning, laboratory investigations, antenatal care, post-natal care, care for new born for up to 3 months, and other healthcare services by neighborhood socioeconomic disadvantage levels. In addition, the result was disaggregated by residence (urban vs. rural). In sum, women of high neighborhood socioeconomic disadvantage status had the least out-of-pocket expenditure for total healthcare utilization, laboratory investigations, antenatal care visits, post-natal care visits, care for new born for up to 3 months, and other healthcare services. However, women from high neighborhood socioeconomic disadvantage status had the greatest out-of-pocket expenditure for family planning service utilization. Based on the results, the out-of-pocket expenditure was significantly more in high neighborhood socioeconomic disadvantage, except in family planning services.

**Table 2:** Prevalence and concentration index (CI) of maternal healthcare services by neighborhood socioeconomic disadvantage level

Indicator	Total healthcare (drugs and services)			Family planning			Laboratory investigations			Antenatal care			Post-natal care			Care for newborn for up to 3 months			Other healthcare		
	I	NI	Total	I	NI	Total	I	NI	Total	I	NI	Total	I	NI	Total	I	NI	Total	I	NI	Total
Quartile 1 (lowest) (%)	45.6	62.5	48.6	1.9	2.1	1.9	17.8	8.6	14.9	2.6	0.3	1.9	1.9	0.7	1.5	1.7	0.1	1.2	19.9	7.7	16.1
Quartile 2 (%)	38.9	56.0	42.7	4.3	1.2	3.2	18.8	8.0	15.3	1.3	0.6	1.1	1.0	0.5	0.8	0.6	0.3	0.5	22.2	8.6	17.5
Quartile 3 (%)	31.8	54.4	37.0	3.5	1.3	2.6	16.1	3.2	11.0	0.4	0.8	0.6	0.4	0.8	0.6	0.3	0.1	0.2	15.1	5.3	11.2
Quartile 4 (highest) (%)	19.8	63.4	27.3	5.0	1.3	3.8	7.1	2.8	5.8	0.1	0.1	0.1	0.2	0.0	0.1	0.1	0.0	0.1	11.0	4.0	8.8
**Total (%)**	**34.2**	**58.6**	**39.1**	**3.6**	**1.5**	**2.9**	**14.9**	**5.6**	**11.7**	**1.2**	**0.5**	**0.9**	**0.9**	**0.5**	**0.8**	**0.7**	**0.1**	**0.5**	**17.1**	**6.4**	**13.4**
Concentration index (CI)	− 0.156	− 0.001	− 0.111	0.151	− 0.092	0.107	− 0.146	− 0.245	− 0.167	− 0.463	− 0.029	− 0.392	− 0.396	− 0.202	− 0.356	− 0.486	− 0.274	− 0.475	− 0.124	− 0.141	− 0.131
Standard error (SE)	0.010	0.012	0.008	0.037	0.083	0.034	0.017	0.041	0.016	0.067	0.144	0.061	0.076	0.139	0.067	0.088	0.279	0.084	0.016	0.038	0.015
*P*	< 0.001	0.923	< 0.001	< 0.001	0.263	0.002	< 0.001	< 0.001	< 0.001	< 0.001	0.838	< 0.001	< 0.001	0.147	< 0.001	< 0.001	0.326	< 0.001	< 0.001	< 0.001	< 0.001
*z*-stat	− 9.93			2.69			2.21			− 2.73			− 1.22			− 0.72			0.42		
*p* value	< 0.001			0.007			0.027			0.006			0.222			0.469			0.677		

*I* Insured/covered by health insurance, *NI* Not insured/not covered by health insurance, *z-stat* test for statistically significant differences in not insured vs. insured concentration index

**Fig. 2:**
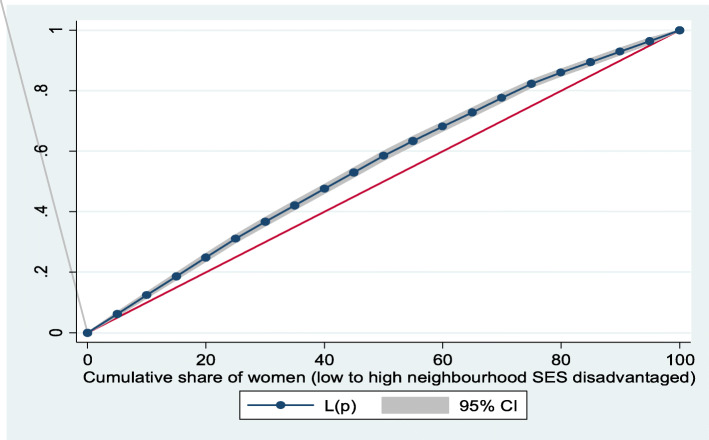
Lorenz curve for overall healthcare utilization

**Fig. 3:**
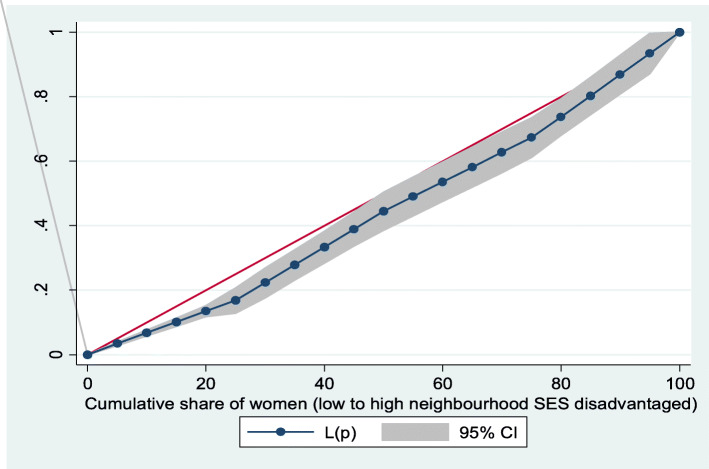
Lorenz curve for family planning utilization

**Fig. 4:**
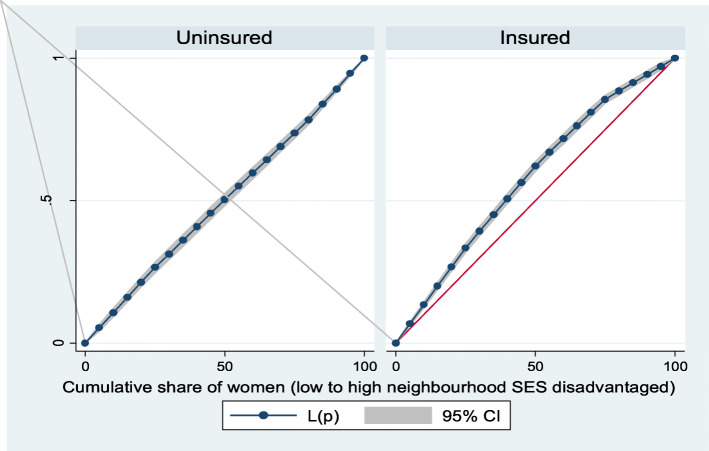
Heath insurance coverage for overall healthcare utilization

**Fig. 5:**
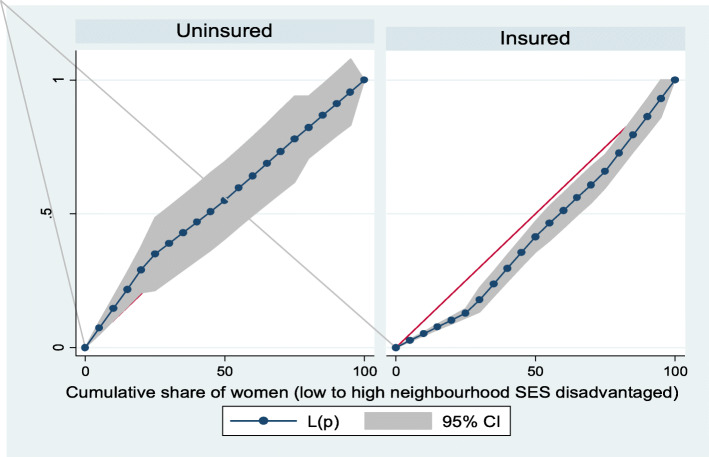
Health insurance coverage for family planning service utilization

Figures 2 and 3 show the neighborhood socioeconomic disadvantage inequalities for women of reproductive age who had out-of-pocket expenditure on overall healthcare and family planning utilization in Ghana. A higher degree of inequalities is confirmed by how far away the curves sag away from the line of equality. Hence, the curves showed that women from low neighborhood socioeconomic disadvantage level had higher for the overall healthcare utilization. The converse was however true for out-of-pocket expenditure in family planning utilization, which means that women from high neighborhood socioeconomic disadvantage level had more family planning service utilization. In addition, disparities in the insured vs. uninsured differentials for out-of-pocket expenditure in the overall healthcare and family planning service utilization are shown in Figs. 4 and 5, respectively.

**Fig. 6:**
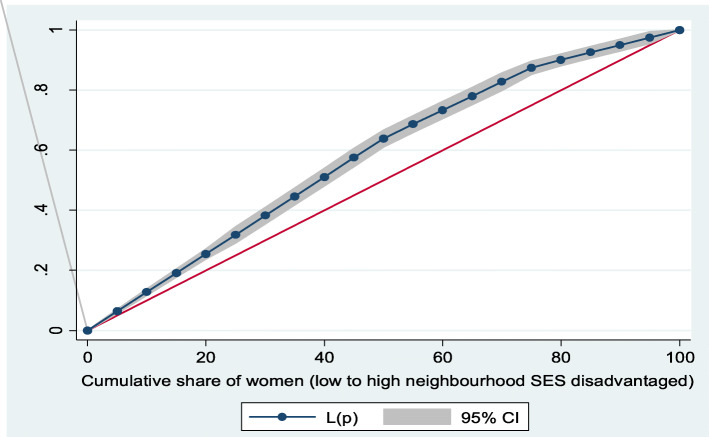
Lorenz curve for laboratory investigations

**Fig. 7:**
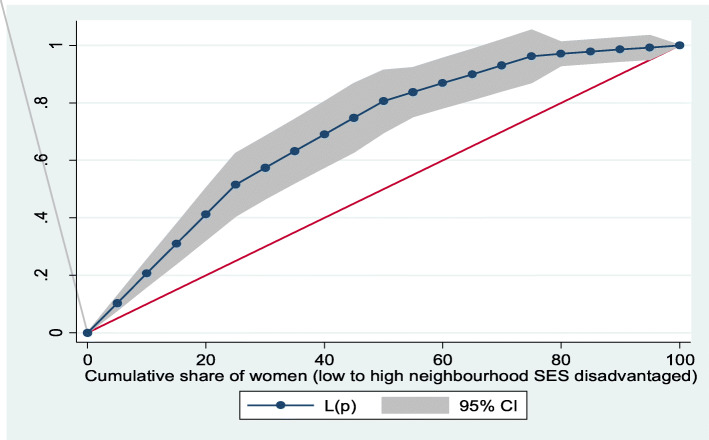
Lorenz curve for antenatal care (ANC) visits

**Fig. 8:**
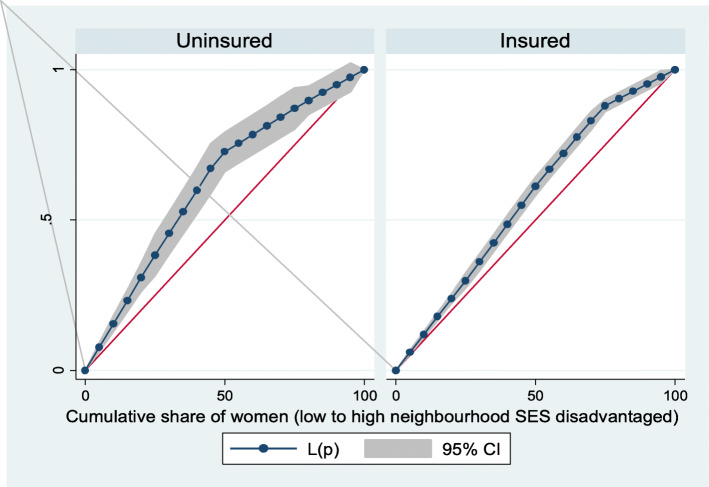
Health insurance coverage for laboratory investigations

**Fig. 9:**
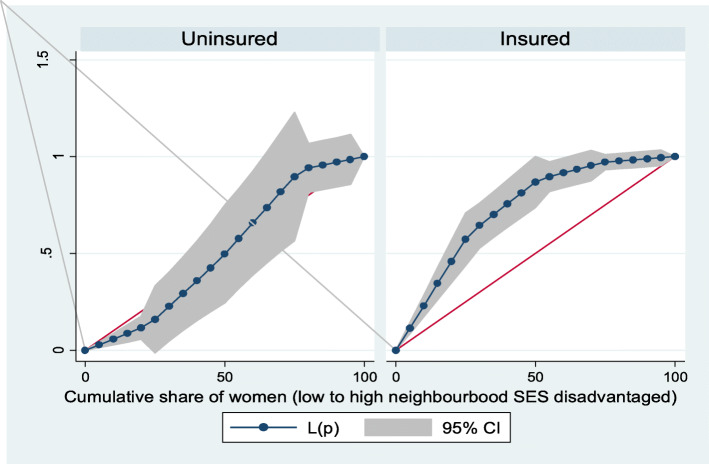
Health insurance coverage for antenatal care (ANC) visits

Based on Figs. 6 and 7, the neighborhood socioeconomic disadvantage inequalities for out-of-pocket expenditure on laboratory investigations and antenatal care utilization showed that women from low neighborhood socioeconomic disadvantage level had higher out-of-pocket expenditure for laboratory investigations and antenatal care utilization. Furthermore, the insured vs. uninsured differentials for out-of-pocket expenditure for laboratory investigations and antenatal care utilization are shown in Figs. 8 and 9, respectively. The farther the curves sag away from the line of equality, the higher the degree of inequalities.

**Fig. 10:**
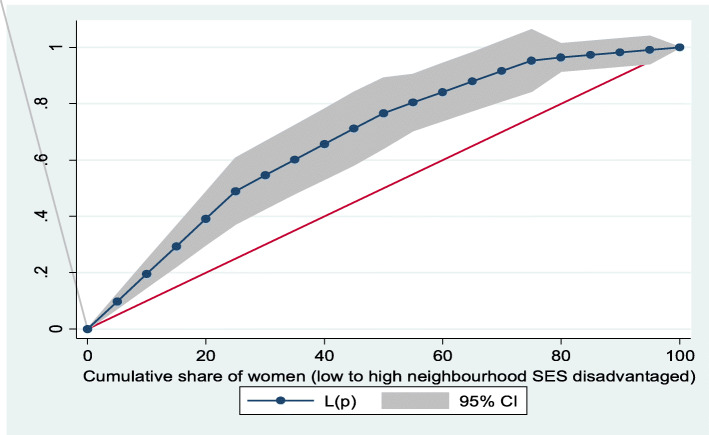
Lorenz curve for post-natal care (PNC)

**Fig. 11:**
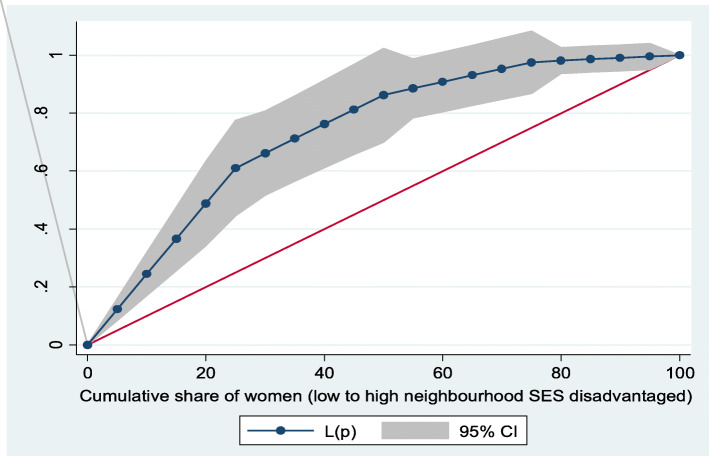
Lorenz curve for care for newborn for up to 3 months

**Fig. 12:**
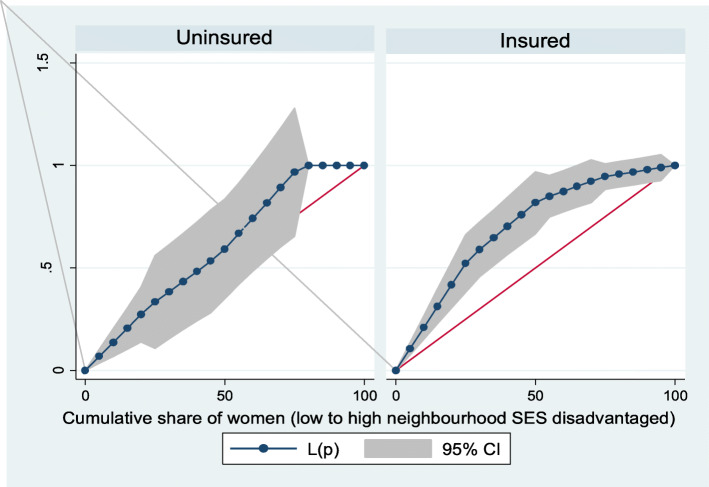
Health insurance coverage for post-natal care (PNC)

**Fig. 13:**
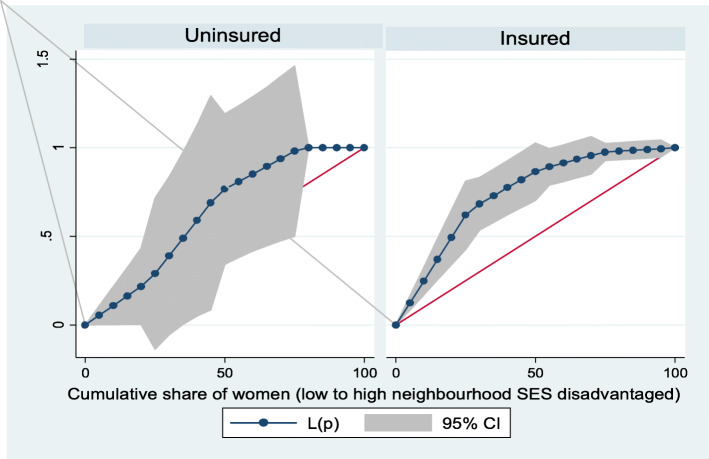
Health insurance coverage for care for newborn for up to 3 months

From Figs. 10 and 11, the neighborhood socioeconomic disadvantage inequalities for out-of-pocket expenditure for post-natal care and care for new born up to 3 months showed that women from low neighborhood socioeconomic disadvantage level had higher out-of-pocket expenditure for post-natal care and care for new born up to 3 months. A higher degree of inequalities is confirmed by how far the curves sag away from the line of equality. In addition, the insured vs. uninsured differentials for out-of-pocket expenditure for post-natal care and care for new born up to 3 months are shown in Figs. 12 and 13, respectively.

**Fig. 14:**
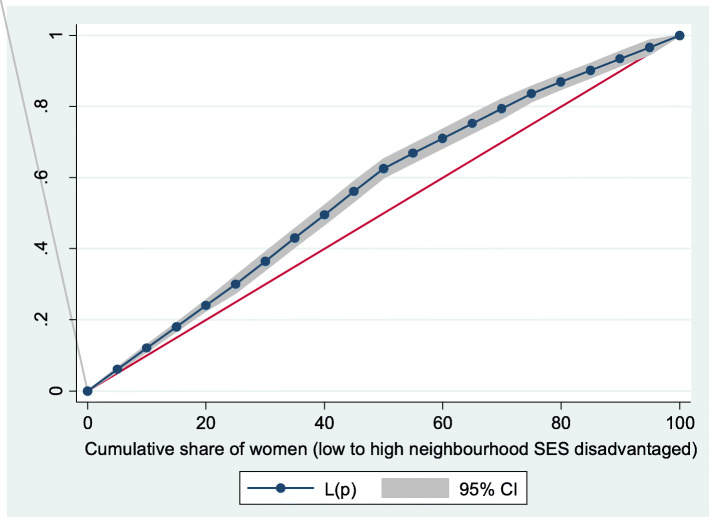
Lorenz curve for other maternal healthcare utilization

**Fig. 15:**
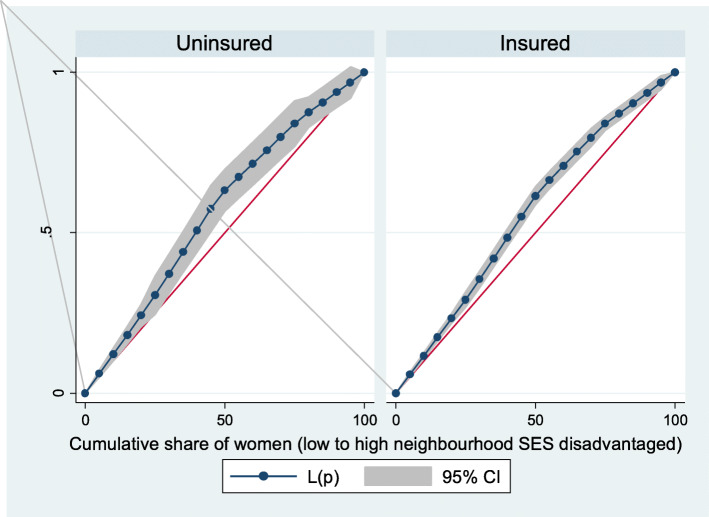
Health insurance coverage for other healthcare utilization

Figure 14 shows the neighborhood socioeconomic disadvantage inequalities for women of reproductive age who had out-of-pocket expenditure on other healthcare service utilization in Ghana. Thus, the curves showed that women from low neighborhood socioeconomic disadvantage level had higher out-of-pocket expenditure for other healthcare service utilization. Notice that the insured vs. uninsured differentials for out-of-pocket expenditure in the other service utilization are shown in Fig. 15. A higher degree of inequalities is confirmed by how far the curves sag away from the line of equality.

## Discussion

To our knowledge, this is the foremost study to examine out-of-pocket health expenditures in relation to neighborhood socioeconomic disadvantage inequalities among women of reproductive age in Ghana. The findings of the study revealed that about two thirds of women are covered by health insurance. This is consistent with reports from a previous study regarding the proportion of the population actively enrolled in the national health insurance scheme [25]. Moreover, our findings on pro-rich out-of-pocket expenditure were consistent with the results from previous studies conducted in resource-poor settings that respondents from higher household wealth quintiles were more likely to have out-of-pocket health expenditures compared with their counterparts from poor household wealth quintiles [26–28]. Further studies are required to investigate the exact reasons why women from low neighborhood socioeconomic disadvantage level in Ghana have higher out-of-pocket health expenditures.

The concept of UHC is to promote health and well-being and to extend life expectancy for all through access to quality healthcare [29]. This presupposes that no one must be left behind. Achieving UHC implies total access to quality healthcare services, including financial risk protection for everyone. NHIS is a mechanism to achieve UHC. The idea of NHIS is that there should be no deductibles, co-payments, or coinsurance or any additional payments at the point of service. Under the health insurance scheme, maternal care benefits include prenatal visits, delivery care, and post-natal visit. Also, care for a child for up to 3 months post-delivery should attract no charges [30]. Unfortunately, the findings from this study as well as from a previous study [31] revealed widespread out-of-pocket health expenditure among the insured. Further studies are needed to investigate the actual causes of out-of-pocket expenditures, especially among the insured. Out-of-pocket expenditure remains a significant barrier to achieving UHC in Ghana and in many resource-constrained settings. In a previous report, even the insured populace had to purchase drugs privately due to lack of stock at health facilities or poor reimbursements of pharmacies by NHIS among other reasons [32]. In a study comparing healthcare expenditures across sub-Sahara Africa, most countries continue to have catastrophic health expenditure, with out-of-pocket expenses ranging between one fifth and over two thirds of total health expenditures, thus indicating that healthcare is still largely unaffordable across the region, despite the advent of health insurance schemes [33].

The finding that women from low neighborhood socioeconomic disadvantage status had increased out-of-pocket health expenditures in most maternal healthcare services than women of high neighborhood socioeconomic disadvantage status is consistent with a previous study [34]. A plausible explanation could be that women of low neighborhood socioeconomic disadvantage status would be expected to have higher service utilization than those of high neighborhood socioeconomic disadvantage status. The results corroborate a previous study that shows pro-rich inequalities in maternal healthcare utilization in Ghana [35]. More importantly, it also underscores global goals that seek to leave no one behind in healthcare use. As indicated, the SDGs outline goals and targets that will help mitigate these inequalities. The findings of this study provide important evidence to highlight the nature of these inequalities. The decomposition results also provide a significant emphasis on the contribution of the NHIS in addressing neighborhood socioeconomic inequality. Moreover, existing evidence suggests that the NHIS has a significant impact on maternal healthcare utilization [36].

Health insurance is a mechanism to reduce the risk of out-of-pocket health expenditure among women who seek maternal healthcare services. Insured patients are still requested to pay for services that should be covered by insurance. The findings of this study point to the fact that implementing UHC will involve scaling up and sustaining the NHIS to reduce inequalities; also, there is a need to ensure efficient operations of the scheme. Other by-way expenses must be prevented at the point of service utilization. For example, though pregnant women may be officially exempted from paying premiums, other unofficial payments that prevent service utilization must be brought to a halt. The fact that inequality still persists in the presence of free pregnancy-related services may be partly due to lack of adequate health facilities. In some hard-to-reach communities, women face the challenge of walking long distances to access healthcare, even though services are free. Consequently, ensuring that health facilities are provided within accessible distances will be a step in the right direction.

### Strengths and limitations

This study used nationally representative data to examine out-of-pocket health expenditure for maternal healthcare service utilization. The use of the data is sufficient to make plausible comparisons among women of reproductive age in Ghana, thereby making it possible to analyze the structure of the out-of-pocket health expenditure and to bring out the health insurance coverage variation. However, the use of secondary data makes it difficult to measure certain salient endogenous variables and the cross-sectional nature of the data requires caution in the use of the findings.

## Conclusion

The out-of-pocket maternal health expenditure was concentrated mainly among women in low neighborhood disadvantage level. Though maternal healthcare services in Ghana are exempted from user charges, the inequalities in out-of-pocket health expenses would create a major hindrance to equitable and universal coverage of maternal healthcare services. The government should strengthen NHIS and use it as a mechanism for increasing maternal healthcare utilization among women in Ghana. There is a need to adopt a holistic approach to measure socioeconomic-related inequalities in healthcare based on the knowledge gap in the assertions underlying the various measurement techniques. In addition, proper regulation should be made to control user charges and to improve the availability of health services; otherwise, health insurance will not be an attractive product. Out-of-pocket health expenditure in turn will be an inhibiting factor in coverage and expansion and particularly will adversely affect the goal of achieving UHC. Ghana’s experience may apply to several resource-constrained settings, particularly the SSA countries as the context for social health insurance is commonly alike.

## Data Availability

Data for this study were sourced from Demographic and Health surveys (DHS) and available here: https://www.dhsprogram.com/data/available-datasets.cfm.

## Abbreviations

AIDS: Acquired immunodeficiency syndrome
CI: Concentration index
DHS: Demographic and health survey
EAs: Enumeration areas
GDHS: Ghana demographic and health survey
HIV: Human immunodeficiency virus
I: Insured
ICF: Inner City fund
LGAs: Local government areas
NHIS: National health insurance scheme
NI: Not insured
PCA: Principal component analysis
SE: Standard error
SHI: Social health insurance
SDGs: Sustainable development goals
TX: Texas
UHC: Universal health coverage
